# Randomized double blind clinical trial evaluating the Ellagic acid effects on insulin resistance, oxidative stress and sex hormones levels in women with polycystic ovarian syndrome

**DOI:** 10.1186/s13048-021-00849-2

**Published:** 2021-07-31

**Authors:** Mahnaz Kazemi, Fatemeh Lalooha, Mohammadreza Rashidi Nooshabadi, Fariba Dashti, Maria Kavianpour, Hossein Khadem Haghighian

**Affiliations:** 1 Department of Nutrition, School of Health, Qazvin University of Medical Sciences, Qazvin, Iran; 2 Department of Obstetrics and Gynecology, Faculty of Medicine, Qazvin University of Medical Sciences, Qazvin, Iran; 3 Department of Pharmacology, School of Pharmacy, Ahvaz Jundishapur University of Medical Sciences, Ahvaz, Iran; 4 Department of Applied Cell Sciences, School of Advanced Technologies in Medicine, Tehran University of Medical Sciences, Tehran, Iran; 5 Metabolic Diseases Research Center, Research Institute for Prevention of Non-Communicable Diseases, Qazvin University of Medical Sciences, Qazvin, Iran; 6 Department of Nutrition, Faculty of Health Qazvin, University of Medical Sciences, Qazvin, Iran

**Keywords:** Ellagic acid, Insulin resistance, Stress oxidative, Anti-mullerian hormone, Polycystic ovarian syndrome

## Abstract

**Objective:**

The design of this study was due to the report of the antioxidant properties of Ellagic acid (EA) for its evaluation on the Insulin resistance (IR), oxidative stress and sex hormones levels in women with polycystic ovarian syndrome (PCOS).

**Methods:**

In this randomized, double-blind, placebo-controlled clinical trial, 60 patients were recruited. Patients were randomly allocated consumed a capsule containing 200 mg of EA per day (*n* = 30) or placebo (*n* = 30) for 8 weeks. The fasting blood sugar (FBS), insulin, IR, total cholesterol (TC), triglycerides (TG), low density lipoprotein (LDL), high density lipoprotein (HDL), total antioxidant capacity (TAC), Malondialdehyde (MDA), C-reactive protein (CRP), Tumor necrosis factor-alpha (TNF-α), sex hormones and anti-mullerian hormone (AMH) were measured at the beginning and end of the study.

**Result:**

At the end of the study, the mean of FBS, insulin, IR, TC, TG, LDL, MDA, CRP, TNF-α, total testosterone, prolactin and AMH were significantly decreased in the intervention group compared to the placebo group (*P* < 0.05). Also, there was a significant increase in the mean of TAC after supplementation with EA (*P* < 0.05). At the end of the study, no significant changes were observed in the mean of anthropometric factors, physical activity and food intake (*P* > 0.05).

**Conclusion:**

EA supplementation can be helpful as a diet supplement in women with PCOS through improvement in insulin resistance. This supplement may be used to reduce metabolic disorders in women.

**Trial registration:**

This study was retrospectively (07–07-2019) registered in the Iranian website (www.irct.ir) for registration of clinical trials (IRCT20141025019669N12).

## Introduction

Polycystic ovary syndrome (PCOS) is a common endocrine disorder that affects about 5–10% of women before menopause [1]. The prevalence of this disorder varies from 2 to 26% in different countries, which can be due to differences in the population under study, the variety of criteria used to define it, the inconsistent cut-off points, and the method used to define each criterion [2]. In the Rotterdam area of the Netherlands, the prevalence is estimated to be up to 20%. The prevalence of this disease in Iran according to Rotterdam criteria is 15.2% [3]. Lack of ovulation or limited ovulation with elevated biological testosterone levels and increased production of ovarian androgens are symptoms of this disorder [4]. In this condition, the patient is more likely to develop insulin resistance (IR), obesity and an increased risk of type 2 diabetes [5]. According the scientific studies, IR can cause oxidative stress condition in these patients. Oxidative stress is effective in increasing the production of androgens, disruption of the stages of development of ovarian follicles and damage of ovarian tissue in patients with polycystic ovary [6]. Oxidative stress indices in patients with PCOS are increased and the total antioxidant capacity of blood is decreased [7]. Also, increased pro-inflammatory cytokines also play an important role in causing systemic IR and thereby worsening the syndrome [8].

The use of antioxidant compounds in reducing IR and chronic inflammation and consequently better management of PCOS syndrome has been of particular interest in recent research [9]. Numerous studies today have demonstrated the impact of non-pharmacological treatments by modifying lifestyle on reproductive performance improvement and reducing cardiovascular metabolic risk factors [10]. Polyphenols as secondary plant metabolites are found in vegetables and fruits. Scientific evidence confirms the beneficial effects of polyphenols in reducing the complications of metabolic diseases. As potent antioxidants, they have protective and therapeutic effects in managing the effects of oxidative stress by regulating inflammatory cytokines and enzymes, enhancing antioxidant defense and suppressing inflammatory pathways and their cellular signaling mechanisms [11]. Ellagic acid is one of the types of polyphenols in which the strong hydrogen bonding network acts as an electron acceptor, which in turn enables EA to participate in a number of reactions. This polyphenol is naturally found in numerous fruits and vegetables, including strawberries, red raspberries, pomegranates and grapes [12]. Ellagic acid can reduce the symptoms of chronic diseases such as dyslipidemia, IR in type 2 diabetes, and nonalcoholic fatty liver disease [13]. Despite advances in information about EA, the mechanism of its activity has not yet been discovered, which may be due to the complexity of its metabolism and depends on various factors. Due to the antioxidant and inflammatory effects that have been reported about EA and the lack of human studies of this polyphenol supplementation in PCOS, the present study aimed to investigate the effect of EA on blood glucose, IR, lipid profile, oxidative stress status, inflammatory factors, sex hormone levels and anti-mullerian hormone in women with PCOS.

## Subjects and methods

### Participants

This randomized, double-blinded, placebo-controlled clinical trial was done on 60 subjects, aged 18–45 years old. In this study, women with PCOS are referred to the Qazvin Kosar Hospital Specialist Center (from 2019–07-15 to 2019–10-20) with study clinical consultant (Gynecologist) meeting the inclusion criteria, the research topic, goals and method of the study were explained, then they received informed consent form if they wish to participate in this study. Women entered the study with at least two of the three Rotterdam criteria [14] to diagnose the syndrome as well as having a Body Mass Index (BMI) of less than 30 kg / m^2^. Patients with a history of abdominal surgery, as well as pregnant and lactating women and those who have been taking supplements in the last three months were not included in the study. Also having underlying illnesses like diabetes, severe psychiatric and behavioral disorders and usage of aspirin, warfarin, heparin and anti-inflammatory drugs (including non-steroids, steroids, antihistamines, and mast cell stabilizers) have been other exclusion criteria. The protocol of the study after approving with the ethic committee of Qazvin University of Medical Sciences (ethic code: IR.QUMS.REC.1398.033), Qazvin, Iran, was registered in the Iranian Registry of Clinical Trials website by the IRCT20141025019669N12 code.

#### Design

All patients met the inclusion criteria were randomly allocated consumed a capsule containing 200 mg of EA per day (*n* = 30) or placebo (*n* = 30) for 8 weeks. The shape, color and size of placebo were similar to the supplement capsule. Supplement was purchased from Supplement Spot and the placebo was made by School of Pharmacy, Tabriz University of Medical Sciences. It should be noted that the effective selective dose for EA supplementation was taken from Falsaperela M et al. [15]. Since oral supplementation with EA has been shown to reduce inflammation (one of the main goals of this research project), this dose was chosen as the dose of choice in this study. Recruitment of patients in this scientific project was done by simple random sampling using a table of random numbers. Based on BMI criteria, participants were divided into two groups using random blocks. According to the double-blind study, the patient, researcher and the specialist physician were blind to the contents of the cans in terms of supplements and placebo. Questionnaires were filled with questions about the basic demographic information and clinical records of the individual and also the participants were evaluated for height and weight.

BMI was calculated by dividing the weight in kilograms by height in meters squared.

To examine more closely the effect of EA supplementation in this study, all patients were advised not to alter their diet and physical activity habits along the study and to avoid foods high in EA. These foods were introduced to people in a list. To control for confounding effects of diet and physical activity, at the beginning and at the end of the study, patients were interviewed by a 3-day dietary recall questionnaire and subjects with moderate physical activity level were enrolled. In this study, relevant questionnaires were filled out to control the diet and physical activity as a confounding factors. Three-day food recalls questionnaire and Nutritionist IV program (San Bruno, CA) modified for Iranian food composition were used to calculate food intake and dietary intake, respectively. Also, the International Physical Activity Questionnaire (IPAQ) was filled out to estimate the amount of physical activity. The conversion of raw data from the IPAQ was done using existing guidelines and converted to metabolic equivalent-minutes/week [16]. Patients were followed up to control their consumption of capsules and prevented from falling out once every 7 days by telephone. In order to fully monitor the use of supplements, participants were asked to hand over a bottle of supplements to the researcher at the end of the study, which would be excluded from the study if used improperly.

#### Laboratory methods

After 10–12 h of overnight fasting, blood samples were collected from patients. Blood samples were taken two to three days after the capsules were taken. Each sample contains 10 cc of blood. Temperature of − 20 °C was used to freeze the serums and then samples were stored at a − 80 °C for future laboratory evaluations. Fasting Blood Glucose (FBS) concentration was measured by the enzymatic method using an Abbot ModelAclyon 300, USA auto analyzer with Pars-Azmone kit (Tehran, Iran). Plasma insulin was measured by using a chemiluminescent immunoassay method (LIAISON analyzer (310,360) Diasorin S.P.A. Vercelli, Italy). HOMA-IR was calculated according to the following formula: HOMA-IR = (fasting insulin (U/ml) × FBS (mg/dl))/405 [17].

Total cholesterol (TC) levels were determined by the enzymatic spectrophotometric method using an auto-analyzer (Abbott, model Alcyon 300, USA) with Pars-Azmoon Kit (Tehran, Iran). Triglyceride (TG) and high-density lipoprotein (HDL) were determined by the enzyme colorimetric method using an automatic analyzer (Abbott, Model Alcyon 300, and USA) with Pars-Azmoon Kit. Low-density lipoprotein (LDL) was calculated by Friedewald formula; LDL (mg/dl) = TC- (HDL-C + TG/5). Serum levels of Total antioxidant capacity (TAC) was measured by a spectrophotometric method using Randox TAS (Laboratories, Crumlin, UK), by an autoanalyzer (Abbott, Model Alcyon 300, and USA). Serum malondialdehyde (MDA) levels were measured by tiobarbituric acid method. Turbidimetric immunoassay was used for measuring of C-reactive protein (CRP) levels (Pars Azmoon kit. Tehran, Iran). Also enzyme-linked immunosorbent assay (ELISA) (DIAsource Co, Belgium) was used for determining serum levels of tumor necrosis factor alpha (TNF-α).

#### Reproductive hormones assay

Serum testosterone and prolactin were assayed using commercial radioimmunoassay kits (Kavoshyar Co., Tehran, Iran). This commercial kit had been previously used with an inter-assay and intra-assay variation of less than 10%. The reference range for testosterone and Prolactin (PRL) are 10 to 35 nmol/l. Luteinizing hormone (LH) was measured by immunochemiluminometric assay, in which intra-assay and interassay coefficients of variation were 3.4% and 3.8%, respectively. The normal LH range is 1.5 to 9.3 IU/l. Follicle-stimulating hormone (FSH) was also measured using immunochemiluminometric assay with an inter-assay and intra-assay coefficient of variation of 3.2% and 6.7%, respectively. The normal FSH range is 1.4 to18.1 IU/l. Serum levels of the Anti-mullerian hormone (AMH) were measured by ELISA (Beckman's kit). The normal range for serum levels of the AMH is 0.08- 16 ng / ml. The mean coefficient of inter-assay and intra-assay for this method was 5.4 and 5.6 percent, respectively.

#### Sample size calculation

The level of the Malondialdehyde factor was used to calculate sample size before and after the administration of pomegranate extract in the study of Hosseini B et al. [18] using the following formula:

$$N=\lbrack(Z_{1-\alpha/2}+Z_{1-\beta})^2(SD_1^2+SD_2^2)\rbrack/\triangle^2$$

Where a (type 1 error) is 0.05, b (type 2 error) is 0.2, S1 and S2 are the variances of MDA, and ∆ represent the difference means of MDA. (MDA before supplementation: 3.3 ± 1, MDA after supplementation: 2.1 ± 0.7). Thus, the power for detecting differences between the 2 groups for various outcomes in the present study was 80%. The sample size was obtained 18 in each group. Considering the drop out in participants during the study, 30 people were considered for each group.

#### Statistical Analysis

Statistical analyses were conducted using SPSS version 20. All data were presented as mean ± SD and were checked for normality by the Kolmogorov–Smirnov test. Due to the normal distribution of variables, the paired sample t-test and the independent sample t-test were applied to analyze differences in variables within and between groups, respectively. The *p* < 0.05 was considered statistically significant.

## Results

**Fig. 1:**
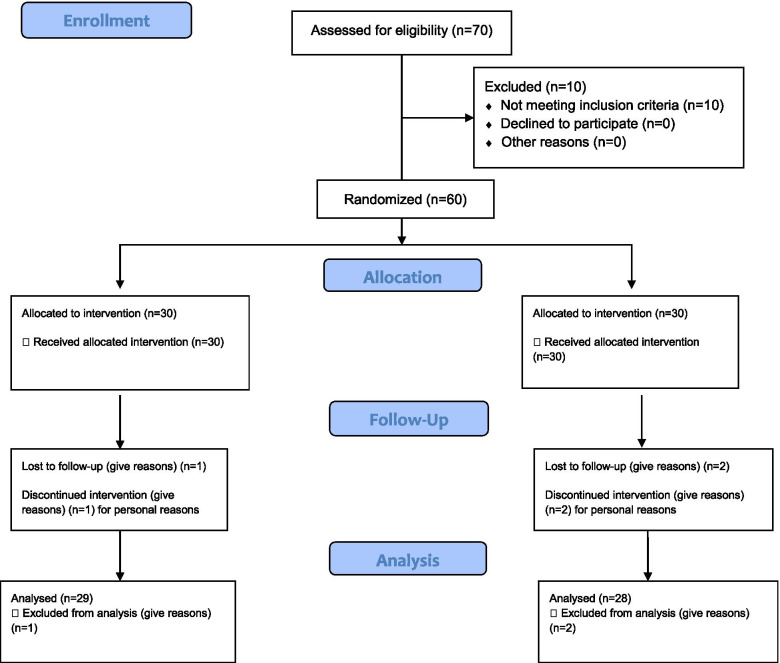
CONSORT 2010 Flow Diagram

Among 70 volunteered patients, ten women dropped out because they did not meet the requirements. A total of 60 people were included in study, and 30 were equally involved in the intervention and placebo groups. During this investigation, two patients of the placebo group and one of the intervention group did not complete the research process and dropped out of the study for personal reasons (Fig. 1). Patient compliance in this study was 95%. The final analysis was done on the subjects who finished the study. The characteristics of the participants are shown in Table 1. There was no statistically significant difference in the baseline characteristics of the participants between the two groups. The mean age of participants in intervention and placebo groups were 25.74 ± 1.19 and 26.09 ± 1.53 years old, respectively (*P* > 0.05). Also, there was no significant difference between the two groups in terms of anthropometric factors in the first study. The mean and standard deviation of weight (70.63 ± 4.15 vs 69.71 ± 5.11 (kg)), Height (162.09 ± 8.33 vs 160.71 ± 9.28 cm) and BMI (26.88 ± 0.59 vs 26.99 ± 0.61 (K g/m^2^)) were in the intervention and placebo groups, respectively. Also, there was no significant difference in the amount of physical activity (37 ± 3.29 vs 36.01 ± 3.5 (met-h/week)) between groups at the beginning of the study (*p* > 0.05; Table 1). It is also noteworthy that at the end of the study, there was no difference in terms of weight, BMI and physical activity between the two groups as well as within the group (*p* > 0.05; Table 1). The mean of energy and macronutrient intake at baseline and the end of the study were shown in Table 2. As shown, there were no statistically significant difference between the groups in terms of average daily intake the energy, protein, fat, saturated fatty acids, unsaturated fatty acids and some micronutrients at the beginning and the end of the study (*P* > 0.05).

**Table 1:** The comparison of baseline characteristics of the participants

Variable		Mean ± SD Placebo(*n* = 28)	Mean ± SD Ellagic acid (*n* = 29)	P1
Age (years, Mean ± SD)	26.09 ± 1.53	25.74 ± 1.19	0.736	Age (years, Mean ± SD)
Height(cm)	160.71 ± 9.28	162.09 ± 8.33	0.77	Height(cm)
Weight (kg)	Before	69.71 ± 5.11	70.63 ± 4.15	0.68
	After	69.2 ± 5.07	70.06 ± 4.51	0.59
	P2	0.651	0.703	
Body Mass Index (K g/m^2^, Mean ± SD)	Before	26.99 ± 0.61	26.88 ± 0.59	0.519
	After	26.79 ± 0.62	26.66 ± 0.64	0.53
	P2	0.864	0.817	
Physical activity (met-h/week)	Before	36.01 ± 3.5	37 ± 3.29	0.218
	After	36.11 ± 3.57	37.61 ± 3.41	0.109
	P2	0.401	0.347	
Metformin dose		1599.03 ± 404.26	1609.11 ± 395.13	0.721

Data are expressed as means ± SD

P1: Comparison of the mean of baseline characteristics between the two groups of Ellagic acid and placebo (Independent samples t-test)

P2: Comparison of mean of baseline characteristics in each group at baseline and end of study (Paired samples t-test)

**Table 2:** The comparison of the dietary intake at the baseline and the end of the study in patients with IBS

**Variables**		Mean ± SD Placebo(*n* = 28)	Mean ± SD Ellagic acid (*n* = 29)	P1
Energy(kcal)	Baseline	2089.95 ± 245.09	2043.57 ± 233.11	0.307
	End	2033.28 ± 237.18	2026.38 ± 292.2	0.413
	P2	0.506	0.367	
Protein(gr)	Baseline	86.42 ± 14.27	84.54 ± 16.41	0.508
	End	82.33 ± 14.09	83.63 ± 16.22	0.419
	P2	0.59	0.546	
Carbohydrate(gr)	Baseline	270 ± 55.2	265.52 ± 50.04	0.502
	End	263.14 ± 50.5	261.33 ± 48.13	0.58
	P2	0.584	0.603	
Fat (gr)	Baseline	73.80 ± 15.71	71.48 ± 14.41	0.714
	End	72.37 ± 15	70.83 ± 14	0.683
	P2	0.633	0.61	
Saturated fatty acids(gr)	Baseline	26.2 ± 8.11	25.67 ± 9.18	0.656
	End	25.32 ± 8.17	24.27 ± 7.03	0.529
	P2	0.57	0.546	
Monounsaturated Fatty acid (gr)	Baseline	22.11 ± 4.77	22.13 ± 3.19	0.807
	End	21.3 ± 4.03	22.17 ± 3.55	0.59
	P2	0.63	0.74	
Polyunsaturated Fatty acid (gr)	Baseline	24.28 ± 7.64	23.18 ± 3.11	0.421
	End	22.9 ± 4.07	22.27 ± 5.17	0.365
	P2	0.29	0.267	
Fiber(gr)	Baseline	10.51 ± 2.2	11.09 ± 3.06	0.129
	End	10.67 ± 2.31	11.43 ± 2.25	0.167
	P2	0.419	0.51	
Vitamin C (mg)	Baseline	71.19 ± 25.69	72.38 ± 15.66	0.758
	End	70.07 ± 24.16	72.29 ± 14.19	0.801
	P2	0.603	0.711	
Vitamin E (IU)	Baseline	10.24 ± 1.89	9.95 ± 2.5	0.309
	End	10.53 ± 1.77	10.07 ± 2.27	0.22
	P2	0.32	0.294	
Selenium (μg/day)	Baseline	119.08 ± 35.63	120.24 ± 37.18	0.86
	End	118.14 ± 38.01	120.18 ± 38.27	0.891
	P2	0.883	0.805	

Data are expressed as means ± SD

P1: Comparison of the mean of dietary intake between the two groups of Ellagic acid and placebo (Independent samples t-test)

P2: Comparison of mean of dietary intake in each group at baseline and end of study (Paired samples t-test)

The effect of EA supplementation on insulin resistance in women with PCOS have been presented in Table 3. As shown in the table, there were no significant differences between these factors at the beginning of the study. In the end of the study, EA reduced the FBS, Insulin and IR, significantly compared the beginning the study (*P* < 0.05). In the placebo group, mean changes in FBS and IR at the end of the study were not significant compared to the beginning study (*P* > 0.05, Table 3). Also the effect of EA supplementation on lipid profile in PCO patients have been presented in Table 4. As shown in this table, there were no significant differences between blood fat components at the beginning of the study. In the end of the study, EA reduced the TC, TG and LDL significantly compared the beginning the study (*P* < 0.05). However, changes in mean HDL at the end of study compared to the first of it, were not significant in the intervention group (*P* > 0.05). The effects of EA oral supplement on stress oxidative status and inflammatory factors in patients were summarized in Table 5. Reduction of MDA, CRP and TNF-α levels in intervention group after supplementation was significant (*P* < 0.05). Also, TAC levels were significantly increased in group that received EA (*P* < 0.05). These differences were not significant in placebo group at the end of the study (*P* > 0.05). As the results of the study show, changes in stress oxidative and inflammatory factors in the intervention group were statistically significant compared to the placebo group at the end of the study (*P* < 0.05, Table 5). Pre- and post-study data on serum sex hormones levels in the two intervention groups and placebo can be seen in Table 6. There were no significant differences in baseline levels of LH, FSH, PRL, total testosterone and AMH between the two groups. In the intervention group, EA supplementation at the end of the study resulted in a statistically significant decrease in total testosterone, PRL and AMH hormone levels compared with the beginning of the study (*P* < 0.05). Changes in the mean of the FSH and LH levels at the end of the study were not significant compared to the beginning of the study (*P* > 0.05). These differences were not significant in placebo group at the end of the study (*P* > 0.05, Table 6).

**Table 3:** Changes in baseline to endpoint measures for insulin resistance in two groups

Variables		Mean ± SD Placebo(*n* = 28)	Mean ± SD Ellagic acid (*n* = 29)	P1
FBS (mg/dL)	Baseline	107.61 ± 20.13	111.17 ± 18.04	0.3
	End	106.17 ± 21.09	94.29 ± 17.43	0.043
	P2	0.622	0.035	
	Mean Changes	-1.44 ± 0.96	-16.88 ± 0.61	0.04
Insulin (µU/ml)	Baseline	14.98 ± 3.07	15.41 ± 3.24	0.327
	End	14.04 ± 2.19	9.63 ± 1.31	0.03
	P2	0.541	0.027	
	Mean Changes	-0.94 ± 0.88	-5.78 ± 1.93	0.041
HOMA-IR	Baseline	3.98 ± 0.85	4.22 ± 1.14	0.272
	End	3.68 ± 0.41	2.24 ± 0.5	0.04
	P2	0.158	0.031	
	Mean Changes	-0.3 ± 0.44	-1.98 ± 0.64	0.043

Data are expressed as means ± SD. *FBS* Fasting Blood Sugar, *HOMA-IR* Homeostatic Model Assessment for Insulin Resistance

P1: Comparison of the mean of insulin resistance between the two groups of Ellagic acid and placebo (Independent samples t-test)

P2: Comparison of mean of insulin resistance in each group at baseline and end of study (Paired samples t-test)

**Table 4:** Changes in baseline to endpoint measures for lipid profile in two groups

Variables		Mean ± SD Placebo(*n* = 28)	Mean ± SD Ellagic acid (*n* = 29)	P1
Chol(mg/dL)	Baseline	192 ± 51.03	197.13 ± 48.71	0.801
	End	190.28 ± 58.51	178.09 ± 45.46	0.024
	P2	0.754	0.0349	
	Mean Changes	-1.72 ± 7.48	-19.04 ± 3.25	0.031
TG (mg/dL)	Baseline	159.19 ± 19.61	163.28 ± 20.55	0.586
	End	157.2 ± 19.29	145.33 ± 16.04	0.042
	P2	0.59	0.04	
	Mean Changes	-1.99 ± 0.32	-17.95 ± 4.51	0.043
HDL (mg/dL)	Baseline	37.08 ± 6.27	36.13 ± 7.7	0.55
	End	37 ± 6.25	39.2 ± 8.02	0.124
	P2	0.61	0.082	
	Mean Changes	-0.08 ± 0.02	3.07 ± 0.32	0.23
LDL (mg/dL)	Baseline	123.08 ± 40.05	128.34 ± 36.89	0.714
	End	122.28 ± 44.11	108.7 ± 35.19	0.04
	P2	0.8	0.031	
	Mean Changes	-0.8 ± 4.06	-19.64 ± 1.7	0.044

Data are expressed as means ± SD. *Chol* Cholesterol, *TG* Triglyceride, *HDL* High-Density lipoprotein, *LDL* Low-density lipoprotein

P1: Comparison of the mean of lipid profile between the two groups of Ellagic acid and placebo (Independent samples t-test)

P2: Comparison of mean of lipid profile in each group at baseline and end of study (Paired samples t-test)

**Table 5:** Changes in baseline to endpoint measures for oxidative stress and inflammatory biomarkers in two groups

Variables		Mean ± SD Placebo(*n* = 28)	Mean ± SD Ellagic acid (*n* = 29)	P1
TAC (mg/dL)	Baseline	1.04 ± 0.03	1.03 ± 0.02	0.41
	End	1.03 ± 0.03	1.91 ± 0.07	0.032
	P2	0.358	0.028	
	Mean Changes	-0.01 ± 0.00	0.88 ± 0.05	0.041
MDA (mg/dL)	Baseline	1.47 ± 0.06	1.51 ± 0.09	0.466
	End	1.45 ± 0.07	0.8 ± 0.03	0.039
	P2	0.611	0.032	
	Mean Changes	-0.02 ± 0.01	-0.71 ± 0.06	0.044
TNF-α (pg/ml)	Baseline	17.01 ± 3.78	16.89 ± 4.02	0.41
	End	16.91 ± 4.04	13.5 ± 3.61	0.033
	P2	0.292	0.03	
	Mean Changes	-0.1 ± 0.26	-3.39 ± 0.41	0.038
CRP(ng/ml)	Baseline	9.37 ± 3.19	9.51 ± 3.37	0.359
	End	9.23 ± 2.87	7.01 ± 2.2	0.045
	P2	0.256	0.033	
	Mean Changes	-0.14 ± 0.32	-2.5 ± 1.17	0.048

Data are expressed as means ± SD. *TAC* Total antioxidant capacity, *MDA* Malondialdehyde, *TNF-α* Tumor necrosis factor alpha, *CRP* C-reactive protein

P1: Comparison of the mean of oxidative stress and inflammatory biomarkers between the two groups of Ellagic acid and placebo (Independent samples t-test)

P2: Comparison of mean of oxidative stress and inflammatory biomarkers in each group at baseline and end of study (Paired samples t-test)

**Table 6:** Changes in baseline to endpoint measures for sex hormones levels in two groups

Variables		Mean ± SD Placebo(*n* = 28)	Mean ± SD Ellagic acid (*n* = 29)	P1
FSH (ng/ml)	Baseline	6 ± 1.85	6.02 ± 1.91	0.57
	End	6.01 ± 1.73	6.07 ± 1.9	0.601
	P2	0.55	0.49	
	Mean Changes	0.01 ± 0.12	0.05 ± 0.01	0.68
LH (ng/ml)	Baseline	10.51 ± 2.19	10.84 ± 2.1	0.601
	End	10.83 ± 2.02	9.73 ± 2.4	0.073
	P2	0.58	0.076	
	Mean Changes	0.32 ± 0.17	-1.11 ± 0.3	0.079
Total Testosterone (ng/ml)	Baseline	0.6 ± 0.031	0.58 ± 0.023	0.61
	End	0.59 ± 0.04	0.38 ± 0.017	0.038
	P2	0.521	0.04	
	Mean Changes	-0.01 ± 0.09	-0.2 ± 0.06	0.042
Prolactin (ng/ml)	Baseline	19.08 ± 4.5	19.91 ± 4.07	0.73
	End	19 ± 4.11	12.3 ± 2.39	0.025
	P2	0.671	0.036	
	Mean Changes	0.32 ± 0.39	-7.61 ± 1.68	0.033
AMH (ng/ml)	Baseline	12.02 ± 3.71	11.27 ± 3.07	0.43
	End	11.9 ± 3.1	7.2 ± 1.01	0.036
	P2	0.318	0.04	
	Mean Changes	-0.12 ± 0.61	-4.07 ± 2.06	0.045

Data are expressed as means ± SD. *FSH* Follicle-stimulating hormone, *LH* Luteinizing hormone, *AMH* Anti-Mullerian hormone

P1: Comparison of the mean of sex hormones between the two groups of Ellagic acid and placebo (Independent samples t-test)

P2: Comparison of mean of sex hormones in each group at baseline and end of study (Paired samples t-test)

### Safety and Adverse Events

No side effects were observed due to the oral administration of EA in any participants. EA resulted in no clinically significant changes in vital signs, urinalyses, serum chemical values or hematological values.

## Discussion

Changing lifestyle and dietary pattern towards sedentary lifestyle and poor nutrition can lead to insulin resistance. Compared to people with normal physiological condition, it is perhaps the most important aspect of IR, the worsening of the disease condition, and a significant increase in mortality [19]. One of the disorders in which IR is a key part of its pathological mechanism is PCOS. The mechanism and main cause of this disorder have not been explained in general, but considering the results of scientific studies, IR, oxidative stress and inflammation are among the first-degree defendants [20]. The aim of this investigation, was to evaluate the EA effects on blood glucose, insulin resistance, lipid profile, oxidative stress status, inflammatory factors, sex hormone levels and anti-mullerian hormone in women with PCOS. After 8 weeks, supplementation with EA, significantly decreased the FBS, IR. Also, at the end of the study, reduction of TC, TG and LDL changes in the intervention group was significant.

Some scientific studies have reported an increase in insulin due to an increase in androgens, and some scientific sources have assumed the exact opposite of this Eq. [21]. However, a decrease in insulin levels and, consequently, a decrease in IR has reduced androgens and better ovarian function [4]. Elevated insulin levels usually cause hyperlipidemia in women with this syndrome. Continuity of these conditions and lack of improvement in biochemical factors can lead to cardiovascular disease [3]. According to the World Health Organization, women with PCOS are more likely to develop myocardial infarction [5]. It seems that the core of all these disorders is IR [22]. The function of genes involved in the secretion and modulation of insulin role, such as genes associated with Sirtuin1 and glucose transporter 2 (Glut2), as well as their effect on insulin signaling, such as glucose transporter 4 (Glut4) in muscle and peroxisome proliferator-activated receptor-gamma (PPARγ) in fat cells, is mainly significantly influenced by dietary polyphenols [23]. EA as a polyphenol and strong antioxidant, has not been studied as a dietary supplement in women with PCOS (according to a search on a scientific database), but its helpful effects on glycemic status have been shown in other metabolic disorders. The clinical trial study of Babaeian et al. [24], that conducted on patients with type 2 diabetes, intervention group drank 240 ml unsweetened pomegranate juice daily. The results of the study showed a significant decrease in insulin resistance at the end of the study, whereas no significant changes were found for serum glucose in this group. Low dose of EA in pomegranate juice or short study time for this dose, may be reasons for the lack of significant effect on glycemic indexes. Esmaeilinezhad et al. [25] investigated the effect of pomegranate juice on cardiovascular risk factors in women with PCOS. Participants received daily pomegranate juice or placebo beverage. Daily consumption of pomegranate juice improved the metabolic outcomes of TG, LDL, HDL and TC, in patients. The possible mechanism of EA that lowers blood cholesterol may be due to its effect on reducing absorption and increasing cholesterol excretion through the feces. The effect of this polyphenol on important and key enzymes in cholesterol metabolism, including hydroxy-methyl-glutaryl-CoA (HMG-COA) reductase and Acyltransferase, has also been reported in laboratory and clinical studies. EA, on the other hand, increases the persistence of beneficial bacteria in the gastrointestinal tract by reducing oxidative stress products, and thus reducing the excess plasma fat by beneficial bacteria can be helpful [26, 27]. On the other hand, the condition that worsens IR, exacerbation of oxidative stress status and increased inflammation in these patients. After glycation reactions and formation of advanced glycation end products (AGEs), production of ROS occurs rapidly increased. This reaction can damage insulin-secreting cells in the pancreas [28]. According to the results of studies, receiving polyphenols can increase the prescription of PPAR-γ and in this way, they can help reduce the chronic complications of PCOS [29]. By summarizing the cellular, experimental, and clinical studies, it can be concluded that relationship between IR and oxidative stress is mutual. In the meantime, inflammation can make both sides of the equation worse [30]. The results of our study indicated that EA significantly improved the stress oxidative index and decreased the inflammatory factors. Abnormalities in oxidative stress index in women with PCOS were reported in the meta-analysis study of Murri et al. [31]. Also, the results of many studies showed high biomarker such as MDA and low indicators of antioxidant system such as TAC in these patients [32]. Goudarzi et al. [33]. investigated the protective effect of EA on sodium arsenic-induced neurotoxicity in rats. They observed that EA administration significantly increased MDA levels, IL1β levels and TNF α levels in the brain compared to the control group. EA administration also increased TAC levels compared to the control group. DNA damage and subsequent harmful genetic changes occur as a result of free radical attacks on DNA. This can lead to DNA methylation and silence of tumor suppressor genes. Therefore, oxidative stress can be a factor in worsening PCOS and even increasing the risk of other metabolic diseases such as cancer in women with this syndrome. One of the pre-inflammatory mediators is nitric oxide (NO), which can cause damage and inflammation due to overproduction. Increased synthesis of the NO synthase enzyme, which is present in macrophages, is increased in PCOS, which can lead to inflammation and increased insulin resistance [34]. In cellular and animal studies, EA has been reported to reduce NO production  [35]. Also, one of the enzymes produced by pre-inflammatory cytokines is the Cyclooxygenase 2 (COX-2) enzyme, which in itself accelerates cascading reactions and releases large amounts of Prostaglandin E2 (PGE2) into the inflamed tissue. However, by inhibiting COX-2 production, inflammation can be reduced [36]. The inhibitory effects of EA on PGE2 production have been reported. Pomegranate-derived polyphenols have also reduced COX-2 production from macrophage cells [37].

Also, EA supplementation resulted in a statistically significant decrease in total testosterone, PRL and AMH hormone levels compared with the beginning of the study. Changes in the mean of the FSH and LH levels at the end of the study were not significant. Hyperandrogenism women usually occur after an increase in insulin [22]. Excessive increase in sex hormones impairs ovulation and increases AMH. Together, these biochemical symptoms will be a prognosis for PCOS [38]. Taking into account the above mechanism and the results of animal and clinical studies, the normalization of the ovulation cycle can be achieved by increasing insulin sensitivity and ultimately reducing sex hormones and lowering AMH [39]. One of the most effective factors in reducing IR is the use of plant polyphenols. So far, no clinical studies have examined the effects of EA polyphenol on mentioned factors, but some micronutrients have been studied with antioxidant properties. The results of Shokrpour et al. [40]. study indicated that receiving CoQ10 in women with PCOS significantly decreased the level of AMH. In this clinical trial, 30 women with PCOS consumed CoQ10 pills 100 mg daily for 3 months. Also, in AbdulameerYahya et al. [41]. study, taking the vitamin D and CoQ10 oral supplement in PCOS patients ameliorated the hormonal profile, oxidative marker, and ovulation outcome. Their results showed that these antioxidants significantly decreased the LH and AMH after eight weeks. Also, studies that have examined the effects of IR-reducing drugs such as metformin in women with this syndrome have also reported a significant reduction in sex hormones such as LH, testosterone and AMH at the end of the study [42]. This clinical study, like other studies, can have strengths and weaknesses. One of the strengths of this study is that for the first time the effect of pure supplement of EA was investigated in women with PCOS. Also, the design of this study as a double-blind randomized clinical trial that had parallel groups, making the results of this study remarkable. It is also important to control confounder factors such as weight, physical activity, and food intake in studies that conducted on metabolic diseases, which was done in this research. However, due to the low budget and the limited number of participants and the duration of the intervention, the results of this study have been statistically analyzed, it should be noted that in order to draw clinical conclusions and examine the clinical effects, it is necessary to conduct studies with a larger number of participants and intervention period.

## Conclusion

In conclusion, the results of this study indicated that 8 weeks of supplementation with EA, 200 mg/day, reduced the levels of blood sugar, blood lipids and IR in PCOS patients. Also, with the ameliorating in the status of oxidative stress and inflammatory status, at the end of the study, we saw a significant decrease in the amount of AMH in these patients. These results provide evidence to support the view that polyphenol antioxidant group with reducing the biochemical factors, can play an important role in helping to control the condition of this syndrome. Nevertheless, further studies are needed to provide additional evidences.

## Data Availability

We make sure that all data and materials support our published claims and comply with field standards.

## Abbreviations

AGEs: Advanced glycation end products
AMH: Anti-mullerian hormone
BMI: Body Mass Index
COX-2: Cyclooxygenase 2
CRP: C-reactive protein
EA: Ellagic acid
ELISA: Enzyme-Linked Immunosorbent Assay
FBS: Fasting Blood Glucose
FSH: Follicle-stimulating hormone
Glut2: Glucose transporter 2
GSH-Px: Glutathione Peroxidase
HDL: High density lipoprotein
HMG-COA: Hydroxy-methyl-glutaryl-CoA
IPAQ: International Physical Activity Questionnaire
IR: Insulin resistance
LDL-C: Low density lipoprotein
LH: Luteinizing hormone
MDA: Malondialdehyde
NO: Nitric oxide
PCOS: Polycystic ovary syndrome
PGE2: Prostaglandin E2
PPARγ: Peroxisome proliferator-activated receptor-gamma
PRL: Prolactin
ROS: Reactive Oxygen Species
TAC: Total antioxidant capacity
TC: Total Cholesterol
TG: Triacylglycerol
TNF-α: Tumor Necrosis Factor Alpha
